# *Streptococcus agalactiae* spondylodiscitis in an immunocompetent adult

**DOI:** 10.1590/0037-8682-0479-2022

**Published:** 2023-02-20

**Authors:** Paula Pires da Costa, Filipa Bacalhau Lima, Raquel Matos Senra

**Affiliations:** 1 Hospital do Divino Espírito Santo de Ponta Delgada, Serviço de Medicina Interna, Ponta Delgada, Portugal.

**FIGURE 1:**
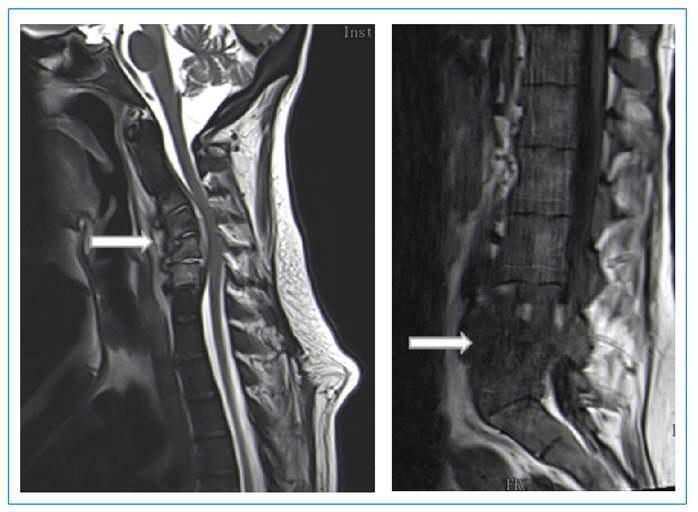
Magnetic resonance imaging of C3-C6 spondylodiscitis, spinal cord compression, and L4-L5 spondylodiscitis (white arrow).

**FIGURE 2:**
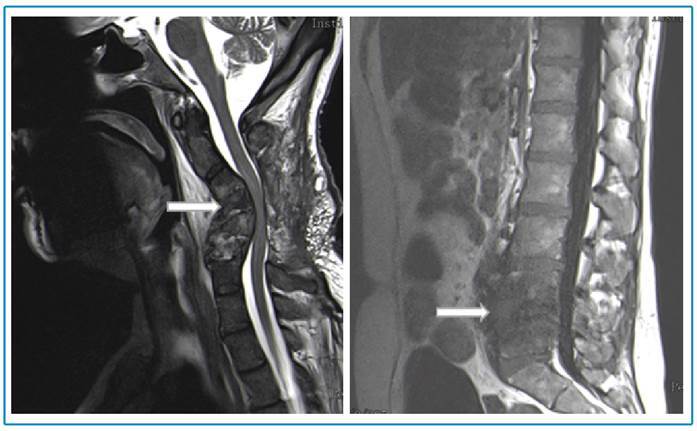
Magnetic resonance imaging reassessment after 7-week antibiotic course (white arrow).

We present the case of a 45-year-old woman without any known underlying diseases or usual medications. The patient presented repeatedly to the emergency department with cervical, dorsal, and lumbar pain, with no symptomatic improvement. Because of clinical worsening, she returned to the hospital with increased and incapacitating pain but no neurological deficit or fever. Laboratory examination on admission revealed a C-reactive protein of 48.9 mg/dL, and a urinalysis suggested a urinary tract infection. Urine and blood cultures were positive for *Streptococcus agalactiae*. Magnetic resonance imaging (MRI) demonstrated C3-C6 spondylodiscitis with an intracanal epidural lesion with severe spinal cord compression, and L4-L5 spondylodiscitis with a small intracanal component (Figure 1). She also had an abscess in the left iliac psoas muscle without any surgical indication. The patient underwent decompression of the epidural space and completed antibiotic therapy with piperacillin/tazobactam followed by ampicillin for 12 weeks. Despite an imaging reassessment showing worsening of the osteomyelitis process (Figure 2), the patient refused orthopedic intervention. She showed clinical improvement with pain control medication and physical therapy. The patient maintained regular follow-ups at the hospital.

Spondylodiscitis most commonly occurs as a result of hematogenous spread from a distant focus^1^. Although *Staphylococcus aureus* is the most common etiologic agent of spondylodiscitis^2^, other microorganisms must be considered. *S. agalactiae* spondylodiscitis is uncommon, especially in immunocompetent patients^3^. This case highlights the importance of clinical suspicion. Patients presenting with neck or lower back pain should be suspected of spondylodiscitis to ensure improved long-term outcomes.
